# Factors related to social disconnectedness among older unpaid caregivers

**DOI:** 10.3389/fpubh.2025.1589103

**Published:** 2025-04-29

**Authors:** Moka Yoo-Jeong, Jonathan Singer, Caroline D. Bergeron, Jodi L. Southerland, Hye Won Chai, Andrew C. Pickett, Yuanjin Zhou, Christian E. Vazquez, Juanita-Dawne Bacsu, Katherine Kwong, Wonkyung Jung, Tiffany R. Washington, Marcia G. Ory, Matthew Lee Smith

**Affiliations:** ^1^School of Nursing, Bouvé College of Health Sciences, Northeastern University, Boston, MA, United States; ^2^Department of Psychological Sciences Texas Tech University, Department of Pharmacology and Neuroscience, Texas Tech University Health Sciences Center, Lubbock, TX, United States; ^3^LIFE Research Institute, University of Ottawa, Ottawa, ON, Canada; ^4^Department of Community and Behavioral Health, College of Public Health, East Tennessee State University, Johnson City, TN, United States; ^5^Institute for Engaged Aging, Department of Psychology, Clemson University, Clemson, SC, United States; ^6^Department of Health and Wellness Design, School of Public Health-Bloomington, Indiana University, Bloomington, IN, United States; ^7^Steve Hicks School of Social Work, University of Texas at Austin, Austin, TX, United States; ^8^School of Social Work, University of Texas at Arlington, Arlington, TX, United States; ^9^School of Nursing, Thompson Rivers University, Kamloops, BC, Canada; ^10^Department of Human Development, Connecticut College, New London, CT, United States; ^11^Connell School of Nursing, Boston College, Chestnut Hill, MA, United States; ^12^College of Social Work, University of Tennessee, Knoxville, TN, United States; ^13^Department of Environmental and Occupational Health, School of Public Health, Texas A&M University, College Station, TX, United States; ^14^Center for Community Health and Aging, Texas A&M University, College Station, TX, United States; ^15^Department of Health Behavior, School of Public Health, Texas A&M University, College Station, TX, United States

**Keywords:** unpaid caregivers, older adults, social environment, financial strain, social disconnectedness

## Abstract

**Background:**

Older unpaid caregivers often face social isolation and loneliness, yet risk factors for social disconnection remain largely unexplored. As the demand for unpaid caregiving rises with an aging population, there is a need for targeted interventions to reduce social disconnectedness in this vulnerable group. This study aimed to identify determinants of social disconnectedness.

**Methods:**

Data came from a sample of 701 unpaid caregivers aged 60 + who completed an internet-based survey assessing sociodemographics, health status, financial strain, social environment, and social disconnectedness. Four sequential regression models were used to identify the unique contribution of these factors related to social disconnectedness.

**Results:**

The first model (*F* = 3.94, *p* < 0.001, a*R^2^* = 0.030) showed that older age (*β* = −0.15, *p* < 0.001), self-identifying as being Black (*β* = −0.10, *p* = 0.008), and higher education (*β* = −0.11, *p* = 0.041) were associated with lower social disconnectedness. Adding health factors in the second model (*F* = 15.33, *p* < 0.001, a*R^2^* = 0.170) revealed that, in addition to age and education, chronic conditions (*β* = 0.12, *p* = 0.001) and possible depression (*β* = 0.35, *p* < 0.001) were associated with social disconnectedness. Including financial strain in the third model (*F* = 15.52, *p <* 0.001, a*R^2^* = 0.212) showed that household income (*β* = −0.10, *p* = 0.012) and financial stress (*β* = 0.18, *p* < 0.001) were additionally associated with social disconnectedness. The final model (*F* = 23.42, *p* < 0.001, a*R^2^* = 0.366) that included social environmental factors showed that age (*β* = −0.07, *p* = 0.033), possible depression (*β* = 0.22, *p* < 0.001), financial stress (*β* = 0.16, *p* < 0.001), and levels of community belonging (*β* = −0.20–0.58, *p* < 0.001) were significantly related to the risk of disconnectedness.

**Conclusion:**

Findings highlight possible intervention targets that have the potential to reduce social disconnectedness among older unpaid caregivers. Particularly, addressing depressive symptoms, reducing financial stress, and enhancing community belonging are essential components to mitigate social disconnectedness risk in this population.

## Introduction

Unpaid caregivers, also known as informal, family, or friend caregivers, play an essential role in supporting individuals with chronic illnesses, disabilities, or age-related conditions (1, 2) by providing care to them without professional training or compensation (3). As such, unpaid caregiving often entails considerable physical, emotional, and financial burdens (1). Many unpaid caregivers dedicate substantial amounts of time providing care, which can limit their ability to engage in social activities, nurture relationships, and participate in community gatherings (4). This risk of social disconnectedness is particularly pronounced for older unpaid caregivers as they face their own age-related challenges, such as physical limitations, declining health, and reduced mobility, which further isolate them from social interactions, leaving them especially vulnerable as they balance their caregiving responsibilities with their personal well-being (5).

Social disconnectedness is the state characterized by a lack of social connection—an umbrella term that encompasses various dimensions of social relationships, including structural (e.g., size of social networks, marital status, living situation, social isolation), functional (e.g., received and perceived social support, loneliness), and quality-related (e.g., satisfaction with relationships, conflict) aspects of an individual’s world (6, 7). Social disconnectedness has a significant impact on health and overall well-being across all age groups (8, 9). The consequences of social isolation and loneliness in caregivers, as well as its influence on the care recipients are well documented (10). While extensive research exists on the risks of social isolation among the older adult population at large, there remains a significant gap in understanding its prevalence and implications among older unpaid caregivers. Furthermore, most studies on social disconnectedness in this group have focused on one aspect of social disconnectedness that generally taps into either structural or functional dimension (11–13) and have not considered diverse aspects of social disconnectedness simultaneously, limiting a comprehensive understanding of the social disconnectedness risk faced by unpaid caregivers.

Caregiving for older adults can negatively affect unpaid caregivers financially due to decreased work hours and household income, high costs of care, and reduced capacity to work (14, 15). A recent review noted that unpaid caregivers face both direct financial costs (i.e., out-of-pocket expenses) and indirect financial costs (i.e., changes in household finances due to reduced labor participation) resulting in significant financial strain on this population (16). Financial strain is a robust predictor of social isolation across the life course, particularly for older adults (17), due to a lack of resources and/or increased labor commitments that result in an inability to engage in leisure and social activities (18). The resulting lack of engagement exacerbates social disconnectedness, creating a vicious cycle of isolation. Given that unpaid caregivers often provide support for extended periods (i.e., years), they are at particular risk for chronic social disconnectedness. However, access to community resources and strong connections within the community can serve as protective factors against the dual burdens of financial strain and social disconnectedness faced by unpaid caregivers.

Social environment plays an important role in health and well-being and is directly related to social disconnectedness (19). Social environment refers to the physical and social contexts in which individuals live, work, or interact, including workplaces, parks, or neighborhoods (including urban, rural or remote areas) (20). This environment influences unpaid caregiver’s lives, including their sense of belonging and access to community resources (21). For example, caregivers in rural or remote areas may have limited access to health care services and community programs, which can exacerbate the effects of an already challenging caregiving role and possibly increase the risk of social disconnectedness.

Understanding the factors that contribute to the risk of social disconnectedness in older unpaid caregivers is crucial for designing targeted interventions that can effectively mitigate isolation and reduce its negative impact on health and well-being. To address this gap in the literature, the purpose of this study was to identify different types of factors associated with social disconnectedness risk among unpaid caregivers aged 60 and older.

## Methods

### Data source

Data were analyzed from a cross-sectional, internet-delivered questionnaire targeting adults ages 60 years and older residing in the United States (22). Participants were recruited nationwide through a Qualtrics Internet Panel (23) between June 2019 and September 2019. To address potential sampling bias introduced by online convenience sampling, quota sampling parameters were employed to diversify the sample across key demographic characteristics, including age, sex, race, and geographic location (22, 24). After participants were identified by Qualtrics, they were presented with a link to the online questionnaire, which required acknowledgment of an Institutional Review Board (IRB)-approved information sheet. Participation in the study was voluntary, and participants could choose to stop taking the survey at any time. Participants were compensated by Qualtrics for their participation, not the research team, in accordance with the Qualtrics Internet Panel policy. A total of 4,101 older adults completed the survey, of which 19 were omitted for missing data on all questions. To align with the study’s focus on unpaid caregivers, participants who indicated “yes” to a question about providing unpaid regular care or assistance to a friend or family member with a health problem or disability were included in the analyses. The resulting analytic sample was 701 unpaid caregivers ages 60 years and older. All survey procedures were approved by the Texas A&M University IRB (IRB2019-0375).

### Variables and measures

#### Sociodemographics

Measures used to identify participant characteristics included self-reported age (i.e., range from 60 to 94 years), sex (i.e., male, female), ethnicity (i.e., non-Hispanic, Hispanic), race (i.e., White or Caucasian, Black or African American, Another Race), education level (i.e., high school education or less, some college, college graduate or more), and whether the participant lived alone (i.e., no, yes).

#### Health factors

Health factors included the number of chronic conditions and possible depression. Participants were asked to report if a healthcare provider told them they had any of 19 chronic conditions (e.g., arthritis, diabetes, high cholesterol, high blood pressure, cancer, chronic pain, osteoporosis, urinary incontinence). The number of self-reported chronic conditions were summed to create a composite score. Depressive symptoms were measured by the two-item version of the Patient Health Questionnaire (PHQ-2) (25, 26). This brief assessment contains the first two items of the PHQ-9 (27), which measures the two cardinal symptoms of depression: depressed mood and anhedonia. The PHQ-2 asks participants to report the frequency they “felt down, sad, or hopeless” and “had little interest or pleasure in doing things” in the past 2 weeks. Response choices were on a 4-point Likert scale that ranged from “not at all” (scored 0) to “nearly every day” (scored 3). These items were summed, with a total score ranging from 0 to 6. The scores were dichotomized using the recommended cutoff of ≥3, indicating those with possible depression (28).

#### Financial strain

Financial strain was assessed using annual household income, current employment status, and a four-item financial stress scale. Participants were asked to self-report their annual household income [i.e., ranging from “less than $10,000” (scored 1) to “$60,001 or more” (scored 6) in $10,000 increments] and if they were currently employed (i.e., “no” or “yes”). Further, participants were asked a series of four items to determine whether they were worried or stressed about having enough money regarding (a) paying their rent or mortgage; (b) buying nutritious meals; (c) buying medications; and (d) meeting their basic needs. Response choices for each item were on a 5-point Likert scale that ranged from “never” to “always.” Based on the frequency distribution, each item was dichotomized as “never/rarely” (scored 0) and “sometimes/usually/always” (scored 1). These four items were summed to create a composite score ranging from 0 to 4 (Cronbach’s alpha = 0.84), with higher scores indicating more financial stress.

#### Social environment

Social environment included rurality/urbanicity of participant’s residence, access to resources, and community belonging. Participants’ county of residence was geocoded based on the 2013 Rural–Urban Continuum Codes (RUCC), which were the most current at the time of data collection. These codes indicate population density within a given county and range from 1 to 9, where higher values indicate residing in more rural areas (29). To capture access to local resources, participants were asked to indicate if “it is easy for me get to appointments, grocery stores, places of worship, and other locations.” Response choices for this item were “yes” (scored 0) and “no” (scored 1), with higher scores indicating more access to resources. Finally, participants were also asked to rate their sense of belonging to their local community, using a 4-point Likert scale. Response choices were “very weak” (scored 1), “somewhat weak” (scored 2), “somewhat strong” (scored 3), and “very strong” (scored 4).

#### Social disconnectedness risk

The primary outcome of interest was the risk of social disconnectedness among participants, measured by the Upstream Social Interaction Risk Scale (U-SIRS-13) (22, 30). This 13-item scale asked participants to report the frequency of feeling disconnected in terms of physical opportunities to interact with others and the emotional fulfillment of such interactions (or lack thereof). Response choices were on a 3-point Likert scale and included “none of the time” (scored 1), “some of the time” (scored 2), and “often” (scored 3). Each item was then dichotomized based on the directionality of the wording to create items scored as “no risk” (scored 0) and “risk” (scored 1). Items were then summed to generate a continuous score from 0 to 13, with higher scores indicating higher risk for social disconnectedness. Cronbach’s alpha for the U-SIRS-13 in the sample was 0.80, which aligns with the strength of reliability coefficients identified in other studies (22).

### Data analysis

All analyses were performed using IBM SPSS Statistics (Version 29). First, descriptive statistics were computed for the sample. Then, we fitted a series of sequential ordinary least squares regression models to examine the relative importance of participants’ sociodemographic characteristics, health factors, financial strain, and social environments on the risk for social disconnectedness. Variable sets were sequentially added as blocks into each subsequent regression model (i.e., a total of four blocks). The proportion of error variance controlled for by each model (i.e., Adjusted R Square) was compared across the four regression models. Regression diagnostics were conducted to assess the assumptions of multivariable linear regression, including the absence of multicollinearity among the variables by checking the variance inflation factor (VIF). For all statistical tests, effects were considered significant at *p <* 0.05.

## Results

Table 1 provides demographic information about participants. The average age was 69.05 (±5.02) years. Most participants were female (63.6%), non-Hispanic (80.0%), and White or Caucasian (71.1%). Most participants reported either having some college education (37.1%) or a college degree or more (46.5%). About 21.0% of participants lived alone. Participants reported 3.47 (±2.57) chronic conditions on average, and 9.6% screened positive for possible depression (PHQ-2 ≥ 3). Participants’ average household income was between $40,000 and $50,000, 79.0% were not employed, and had low worry/stress about money (i.e., average score of 1.08). Participants primarily resided in metropolitan areas (i.e., average RUCC of 1.93), with 92.4% reporting being able to easily get to appointments, grocery stores, places of worship, and other locations. About 31% of participants reported having a “somewhat weak” or “weak” sense of belonging to their local community.

**Table 1:** Sample characteristics (*n* = 701).

Variable	% or Mean (±SD)
Upstream Social Interaction Risk Scale (range: 0 to 13)	3.57 (±2.76)
Sociodemographic factors	
Age (range: 60–94 years)	69.05 (±5.02)
Female	63.6%
Male	36.4%
Non-Hispanic	80.0%
Hispanic	20.0%
White or Caucasian	71.1%
Black or African American	21.5%
Another Race	7.4%
Highest Level of Education: High School or Less	16.4%
Highest Level of Education: Some College	37.1%
Highest Level of Education: College Graduate or More	46.5%
Live Alone: No	79.3%
Live Alone: Yes	20.7%
Health factors	
Number of Self-Reported Chronic Conditions (range: 0–18)	3.47 (±2.57)
PHQ-2 for Depression Symptoms: Scores 0–2	90.4%
PHQ-2 for Depression Symptoms: Scores 3–6	9.6%
Financial strain	
Annual Household Income (in ~$10,000 increments)	5.03 (±1.94)
Employed: No	79.0%
Employed: Yes	21.0%
Financial Stress Scale (range = 0–4)	1.08 (±1.45)
Social Environment	
RUCC for Residential Rurality (range: 1–9)	1.93 (±1.62)
Ease Getting to Appointments and Other Locations: Yes	92.4%
Ease Getting to Appointments and Other Locations: No	7.6%
Sense of Belonging to Local Community: Very Weak	6.7%
Sense of Belonging to Local Community: Somewhat Weak	24.8%
Sense of Belonging to Local Community: Somewhat Strong	46.6%
Sense of Belonging to Local Community: Very Strong	21.8%

Table 2 reports findings from the sequential ordinary least squares regression models. The first model (Model A: sociodemographic factors only; *F* = 3.94, *p* < 0.001, a*R^2^* = 0.030) showed that each additional year of age (*β* = −0.15, *p* < 0.001) and self-identifying as being Black/AA (*β* = −0.10, *p* = 0.008) were associated with lower risk for social disconnectedness. Participants who attended some college (*β* = −0.15, *p* = 0.007) or had college education or more (*β* = −0.11, *p* = 0.041) had lower risk for social disconnectedness compared to high school or less than high school levels of education.

**Table 2:** Ordinary least squares regression models.

	Model A				Model B				Model C				Model D			
Variable	95% CI				95% CI				95% CI				95% CI			
	Beta	*P*	Lower	Upper	Beta	*P*	Lower	Upper	Beta	*P*	Lower	Upper	Beta	*P*	Lower	Upper
Age	−0.15	<0.001	−0.12	−0.04	−0.13	<0.001	−0.11	−0.03	−0.10	0.004	−0.10	−0.02	−0.07	0.033	−0.07	0.00
Male (vs. Female)	0.04	0.353	−0.23	0.64	0.03	0.354	−0.21	0.59	0.05	0.198	−0.13	0.65	0.01	0.702	−0.28	0.42
Hispanic (vs. Non-Hispanic)	−0.01	0.752	−0.63	0.46	−0.04	0.308	−0.77	0.24	−0.05	0.143	−0.86	0.13	−0.03	0.304	−0.68	0.21
Black or African American (vs. All Other Races)	−0.10	0.008	−1.21	−0.19	−0.07	0.050	−0.95	0.00	−0.11	0.002	−1.21	−0.27	−0.05	0.111	−0.78	0.08
Another Race (vs. All Other Races)	−0.03	0.524	−1.09	0.56	−0.01	0.843	−0.84	0.69	0.00	0.901	−0.79	0.70	−0.02	0.643	−0.83	0.51
Some College Education (vs. All Other Levels)	−0.15	0.007	−1.43	−0.22	−0.15	0.002	−1.44	−0.32	−0.10	0.037	−1.15	−0.04	−0.08	0.084	−0.94	0.06
College Education or More (vs. All Other Levels)	−0.11	0.041	−1.20	−0.02	−0.11	0.032	−1.14	−0.05	−0.03	0.558	−0.74	0.40	0.00	0.932	−0.53	0.49
Lives Alone (vs. Lives With Others)	0.03	0.395	−0.29	0.73	0.03	0.418	−0.28	0.66	−0.01	0.864	−0.51	0.43	0.02	0.550	−0.30	0.56
Number of Self-Reported Chronic Conditions					0.12	0.001	0.05	0.20	0.08	0.028	0.01	0.16	0.05	0.093	−0.01	0.12
Depression Symptoms 3 + (vs. Scores of 0–2)					0.35	<0.001	2.61	3.90	0.30	<0.001	2.19	3.47	0.22	<0.001	1.48	2.66
Annual Household Income Level									−0.10	0.012	−0.26	−0.03	−0.05	0.202	−0.17	0.04
Not Employed (vs. Employed)									0.00	0.997	−0.47	0.47	0.02	0.539	−0.29	0.56
Financial Stress Scale									0.18	<0.001	0.20	0.48	0.16	<0.001	0.18	0.44
RUCC for Residential Rurality													0.02	0.438	−0.06	0.15
Ease Getting to Appointments and Other Locations													0.06	0.060	−0.03	1.28
Somewhat Weak Belonging to Local Community (vs. All Other Levels)													−0.20	<0.001	−1.98	−0.55
Somewhat Strong Belonging to Local Community (vs. All Other Levels)													−0.48	<0.001	−3.32	−1.93
Very Strong Belonging to Local Community (vs. All Other Levels)													−0.58	<0.001	−4.65	−3.14
	Adjusted R Square = 0.030				Adjusted R Square = 0.170				Adjusted R Square = 0.212				Adjusted R Square = 0.366			

The second model added health factors to the analysis (Model B: sociodemographics + health factors; *F* = 15.33, *p* < 0.001, a*R^2^* = 0.170) and showed that age and education remained significant predictors of lower social disconnectedness risk. Further, self-reported chronic condition (*β* = 0.12, *p* = 0.001) and having possible depression (*β* = 0.35, *p* < 0.001) were additionally associated with higher risk for social disconnectedness.

The third model (Model C: sociodemographics + health factors + financial burden; *F* = 15.52, *p <* 0.001, a*R^2^* = 0.212) showed that, in addition to the significant findings from Models A and B, higher annual household income was associated with lower risk for social disconnectedness (*β* = −0.10, *p* = 0.012), whereas being more worried or stressed about money was associated with higher risk for social disconnectedness (*β* = 0.18, *p* < 0.001).

The final model (Model D: sociodemographics + health factors + financial burden + social environment; *F* = 23.42, *p* < 0.001, a*R^2^* = 0.366) showed that age (*β* = −0.07, *p* = 0.033), possible depression (*β* = 0.22, *p* < 0.001), and financial stress (*β* = 0.16, *p* < 0.001) remained significantly associated with higher social disconnectedness. Compared to those with very weak belonging to their local community, participants who reported somewhat weak (*β* = −0.20, *p* < 0.001), somewhat strong (*β* = −0.48, *p* < 0.001), or very strong (*β* = −0.58, *p* < 0.001) community belonging had lower risk for social disconnectedness.

**Figure 1:**
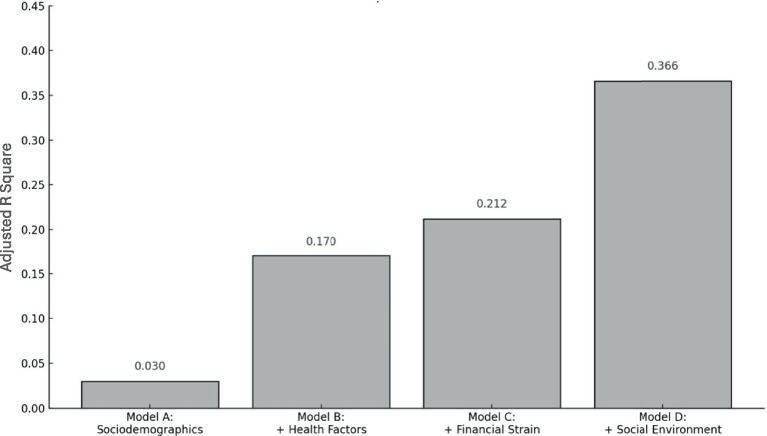
Sequential contribution to explained variance in social disconnectedness. Bar chart displays adjusted R Square values for each sequential regression model. Model A includes sociodemographics; Model B adds health factors; Model C adds financial strain; and Model D adds social environment variables. Each block meaningfully increases the model’s explanatory power, with the final model accounting for 36.6% of the variance in social disconnectedness.

Figure 1 illustrates the additive contributions of each block of variables in terms of model fit (i.e., higher Adjusted R Square values indicate stronger models in that that the included variables explain more variance in social disconnectedness) for Models A through D (i.e., starting with sociodemographic characteristics and sequentially adding, health factors, financial strain, and social environment variables). Each block meaningfully increases the model’s explanatory power, with the final model accounting for 36.6% of the variance in social disconnectedness.

## Discussion

This study identified the unique contributions of sociodemographic, health, financial, and social environmental factors to social disconnectedness risk among older unpaid caregivers aged 60 and above. The findings underscore critical areas for both prevention and intervention strategies to mitigate social disconnectedness in this population. One of the most significant findings was the strong and consistent association between probable depression and increased risk of social disconnectedness across all three models in which it was included. This finding aligns with previous research indicating that mental health, such as depression, can exacerbate feelings of isolation and hinder the ability to engage in social activities (31, 32). Depressive symptoms may lead to withdrawal from social networks, reduced motivation to seek social support, and a diminished sense of belonging (32). Given that caregivers often experience emotional strain due to their caregiving responsibilities, it is critical for health care professionals to screen for depressive symptoms early and address depression using evidence-based interventions, such as behavioral activation (33), and cognitive-behavioral therapy (34, 35). However, caregivers are often “hidden patients” making it difficult for healthcare providers to identify them and intervene on their behalf. To address this concern, Holiday and colleagues (2022) developed the C.A.R.E. framework (36) – Caregiver well-being, Advanced care planning, Respite, and Education—which is designed to educate clinicians about the need to support family caregivers and connect them to interventions and support. Dissemination of the C.A.R.E. framework could enhance clinicians’ efforts to screen for depression and social disconnectedness in this population. Ultimately, reducing depressive symptoms could result in improved emotional well-being, greater engagement in social interactions, and reduced social disconnectedness risk.

Financial stress was another significant factor associated with social disconnectedness risk among unpaid caregivers. The financial burden associated with caregiving responsibilities, including out-of-pocket costs such as health care expenses for care recipients, can significantly limit the caregivers’ ability to use resources for participating in social activities to maintain connections (15). This financial strain is particularly pronounced among spousal caregivers, who are more vulnerable compared to adult children caring for parents. This is important to highlight particularly among older caregivers who are retired and reliant on fixed incomes (37). Further, caregiving responsibilities may reduce working hours for unpaid caregivers who are still in the workforce (38). Strategies to address financial stress should extend beyond providing financial assistance to include employment support programs for unpaid caregivers to help them balance work and caregiving responsibilities. As individuals increasingly remain in the workforce beyond traditional retirement age, sustaining employment may provide caregivers with financial stability, social engagement opportunities, and a sense of purpose. Promoting caregiver-inclusive institutional policies (e.g., such as flexible work hours, expanded caregiving leave, and remote work options) may play a critical role in mitigating financial stress and preserving social connection, especially among those providing ongoing unpaid care while working. In addition, providing free or low-cost respite services could allow unpaid caregivers to engage in more social activities or re-enter the workforce, which can mitigate the risk of social disconnectedness.

A strong sense of community belonging was identified as a significant factor mitigating the risk of social disconnectedness among older unpaid caregivers, underscoring the importance of social integration and community involvement in reducing isolation within this population. Research demonstrates that a sense of belonging to one’s community may serve as a protective buffer against caregiving burden (39) through collective efforts in problem solving and inducing a greater sense of identity (40). Community-based interventions that promote social inclusion, such as neighborhood programs, support groups, and local activities, could foster a stronger sense of belonging and reduce the risk of social disconnectedness. Interpersonal strategies such as peer mentoring may help reduce isolation and foster a sense of community, which could help reduce social disconnectedness risk (41, 42). Technology-based interventions may help to facilitate social participation for caregivers who have impaired physical functioning or no longer drive, as well as for individuals who are in a high intensity caregiving context (43). Taking an upstream approach to intervention by targeting unpaid caregivers that are living in certain high risk social environments, such as those in isolated areas or with limited access to resources, is critical.

Caregivers who self-identify as Black or African American had reduced risk of social disconnectedness. This contrasts with existing literature that shows higher levels of social isolation in racial and ethnic minorities due to structural inequities and discrimination (44) but aligns with the evidence on protective effects of being a racial/ethnic minority caregiver through increased access to social support (45). Caregivers who self-identify as Black or African American may benefit from stronger ties to family, friends, fictive kin, and community (46) which provide emotional and social support that enhances social connections. Previous studies suggest that Black or African American communities may have more robust unpaid caregiving networks and a cultural emphasis on collective support, which can foster a sense of community (45). However, this finding warrants further exploration to better understand the complex interplay between race, caregiving, and social connection. It also highlights the need for culturally tailored interventions that build upon existing strengths within communities (47).

Several limitations should be considered when interpreting the findings. First, the study relied on a non-probabilistic, internet-based sample, which may introduce selection bias. While the study attracted participants from a range of sociodemographic backgrounds, participants with internet access and the ability to complete online surveys may not fully represent the broader population of older unpaid caregivers, particularly those who are more socially isolated or economically disadvantaged. This study may have also excluded unpaid caregivers experiencing higher levels of burden who lacked the time or capacity to engage in this study. Similarly, the sampling methodology may have inadvertently excluded individuals who are less digitally connected—such as those with limited internet access, lower digital literacy, or greater social isolation. These individuals may represent a particularly vulnerable subgroup of caregivers who face compounded barriers to social engagement and support. As a result, our findings may underestimate the true prevalence or severity of social disconnectedness among the broader population of older unpaid caregivers. Future research should consider using mixed-modal recruitment strategies to ensure inclusion of digitally disconnected individuals and to more comprehensively assess social risks across the caregiving spectrum. Second, we did not collect information about the care recipients or specific caregiving contexts, including the types or intensities of caregiving. For example, caring for an individual with Alzheimer’s disease or related dementias differs significantly from caring for someone with mobility challenges. As such, the potential influence of these distinct caregiving contexts on the risk of social disconnectedness among unpaid caregivers warrants further investigation. Future research should incorporate more detailed measures of caregiving roles and responsibilities to better understand how specific caregiving contexts contribute to social disconnectedness among older unpaid caregivers. Differentiating between caregiving subtypes may also help tailor interventions to those at greatest risk of social isolation. Third, the study did not assess whether participants were the primary caregivers or part of a broader caregiving network. Understanding whether additional caregivers co-provide care could offer valuable insights into how caregiving networks influence social connectedness. Fourth, the cross-sectional design of the study limits the ability to infer causal and direction of the relationships between identified risk factors and social disconnectedness. Longitudinal studies are needed to clarify the directionality and temporal dynamics of how sociodemographic characteristics, health factors, and caregiving contexts influence social connectedness as older unpaid caregivers age. Lastly, since the current study used the PHQ-2 to assess depressive symptoms (27), our findings should be interpreted with caution, recognizing that they reflect screening-level indicators rather than formal diagnoses.

## Conclusion

This study contributes to the growing body of knowledge on social disconnectedness risk among older unpaid caregivers by identifying key risk factors reflecting intervention targets. Through sequential regression modeling, this study assessed the unique contributions of various factors related to social disconnectedness risk in older unpaid caregivers. Addressing depressive symptoms, reducing financial stress, and enhancing community belonging are essential components to mitigate social disconnectedness risk in this population. Given the projected growth of older unpaid caregivers, this research highlights an urgent need to provide resources and services that promote social connection, strengthen supportive networks, and improve overall well-being among older unpaid caregivers.

## Data Availability

The raw data supporting the conclusions of this article will be made available by the authors, without undue reservation.
